# Application of artificial intelligence in lung cancer diagnosis, therapy, and prognosis

**DOI:** 10.1186/s12920-026-02360-3

**Published:** 2026-04-10

**Authors:** Jianhua Wang, Ran Tian, Jianting Liu, Saizhu Duan, Yingsong Zhang, Shuxian Guo

**Affiliations:** 1 Department of Oncology, The First Affiliated Hospital of Dali University, Dali, 671000 China; 2 Shanxi Medical University, Taiyuan, 030001 China

**Keywords:** Artificial Intelligence, Lung cancer, Diagnosis, Therapy, Prognostic

## Abstract

Lung cancer is the highest global incidence and mortality rates, imposing substantial burdens on patients’ lives. As the most transformative technology of the modern era, artificial intelligence (AI) has been deeply integrated into medical research and clinical practice. Currently, AI is widely applied in the diagnosis, treatment, and prognostic evaluation of lung cancer. This paper outlines the historical development of lung cancer and its current diagnostic and therapeutic approaches. Then examines the evolution of AI technologies and their learning methodologies, culminating in a focus on the latest advancements in AI applications for lung cancer diagnosis, treatment, and prognosis. This article summarizes the current state of AI integration in lung cancer care and proposes potential directions for future development.

## Introduction

Cancer posed a difficult issue for society, public health, and the economy in the current century. Lung cancer, making up one in eight cancer cases worldwide, also known as primary bronchial cancer, originates from the trachea and mucous membranes or glands of the bronchi, and bronchi is the most common lung malignancy. With nearly 2.5 million new cases in 2022, lung cancer was the most diagnosed cancer. It is also the leading cause of cancer fatalities, with an estimated 1.8 million deaths [1]. Tobacco represents the most prevalent cause of cancer mortality. It is estimated that one cancer death is attributable to every 3 million cigarettes smoked [2], with additional contributions from alcohol consumption [3], 2and exposure to secondhand or passive smoking [4]. Occupational exposure [5] and air pollution [6] are also common causes of lung cancer [7]. Lung cancer can be divided into two major groups: small cell lung cancer (SCLC) and non-small cell lung cancer (NSCLC). NSCLC includes lung adenocarcinoma (LUAD), lung squamous cell carcinoma (LUSC), large cell carcinoma, and bronchial carcinoid tumors. About 85% of primary lung cancer cases are NSCLC, and most patients have advanced, non-resectable disease at diagnosis [8]. Currently, various treatment options are available [9], including surgical treatment when the patient’s condition is stable, radiation therapy [10], chemotherapy [11], and other combined therapy treatments [12–14]. There are also targeted drug treatments for patients with poorer conditions, including immunotherapies such as CAR-T cells [15]. These treatments can temporarily prolong the survival time of patients, but they cannot currently cure cancer. Therefore, there is still significant room for improvement in the treatment and research of lung cancer.

AI is the capacity of a system to accurately analyze external data, learn from it, and utilize those insights to meet specific goals and tasks with adaptable strategies. AI is a fundamental aspect of computer science that has spread across all fields of science and technology, ranging from engineering to healthcare. As society enters the information age, science and technology are developing rapidly. From the first computer to the current big data, it is inseparable from many technologies and their promotion. From the initial Google to the current Deepseek and other forms of AI emergence, it not only promotes the progress and development of society but also facilitates people’s daily life, and at the same time, promotes leading enterprises, medicine, and other industries. The integration of AI and medicine has promoted the development of medical informatics and biomedical engineering, and an increasing number of people have begun to pay attention to AI in the fields of scientific research, medicine, and teaching [16]. AI has driven advancements in the precision treatment of various diseases, such as diabetic retinopathy [17], latent tuberculosis infection [18], gingivitis and periodontal disease [19], and cancer [20]. AI has also made significant progress in guiding and researching clinical pharmacotherapy, such as drug design [21], drug response [22], virtual screening [23], predicting drug-target or drug‒drug interactions [24], and drug pharmacovigilance [25]. Additionally, in the direction of Chinese medicine, owing to the small number of natural molecular compounds and the diversity of drug discoveries, the innovation of AI in the field of virtual screening can help improve the efficiency and accuracy of new drug discovery [26].

## Lung cancer

### History of lung cancer

In 1815, lung cancer was identified as a distinct condition following the publication of Laennec’s work ‘Encephaloides’, named for the tumor’s similarity to brain tissue, in the dictionary of Medical Science. In 1855, Thomas Balfour [27] published the first case of encephaloid cancer of the right kidney and of the lung. In 1874, Elliott [28] documented the course of the disease in patients for the first time, naming it primary lung cancer. In 1912 [29], British doctors performed the world’s first lobectomy due to a tumor in a 44-year-old male patient. In the same year, Isaac Adler [30] published the first literature review concerning lung cancer. Since then, the public has become aware of lung cancer. In the early development of lung cancer treatment, although efforts were devoted to this field, limited progress had been made [31], with surgical interventions such as pneumonectomy [32] and lobectomy [33, 34], representing the only available options at that time. In the subsequent development of medicine, the application of TNM staging in lung cancer has become increasingly widespread [35, 36]. In recent years, diagnostic technology in clinical imaging and pathological diagnosis has undergone significant advancements. The approach to treating lung cancer has become more precise, and personalized treatment has been developed with the advent of immunotherapy.

### Diagnosis of lung cancer

Lung cancer often leads to death because it is usually not detected until it has progressed significantly. Early detection is vital, in many countries, lung cancer screening is now a guideline-recommended tool. Currently, low-dose CT (LDCT) increases the likelihood of detecting small non-calcified nodules and, thus, lung cancer at an earlier and more curable stage. It is routinely used for lung cancer screening [37]. With the advancement of technology, there has also been significant progress in detection methods, which are also used for screening [38] (Table 1).

**Table 1:** Different methods for detecting lung cancer. Each diagnostic method has advantages and disadvantages, this comparison will facilitate better decision-making for doctors in subsequent diagnoses

Method	Basic principle	Advantage	Disadvantage	Inference
Computed tomography	Different attenuation of X-rays as they pass through various body tissues	Convenient, fast, imaging data inside the body in detail	Ionizing radiation may increases 5% of cancer cases each year	[39–41]
Magnetic resonance imaging	The changes of atomic nuclei in a magnetic field	Nonionizing radiation, Low susceptibility; Lower specific absorption rates	Inability to identify calcification foci, Long transverse relaxation times	[42]
Positron emission tomography	Differential manifestations of PET imaging agents or their metabolic molecules in vivo	Noninvasive visualization, characterization, quantification, image function metabolism	Low detection rate for small lesions less than 5 mm	[43]
Sputum Examination	Different numbers of nucleic acids and proteins	Noninvasive, easy to obtain; repeatedly extracted	Low accuracy rate	[44]

### Therapy

Currently, the treatment of lung cancer has significantly improved, including advances in radiation therapy (RT), along with stereotactic ablative radiotherapy (SABR), as well as new targeted therapies and immunotherapies. Various cancer therapies have effectively prolonged the survival time of patients. For early-stage resectable NSCLC, adjuvant immunotherapy significantly improves disease-free survival [45, 46]. It could strengthen the immune response, better pathological outcomes, and decrease treatment delays [47]. For unresectable stage III NSCLC, concurrent chemoradiotherapy or immunotherapy is standard care. Pembrolizumab, nivolumab, and atezolizumab are currently the three immune checkpoint inhibitors approved for use in first- and/or second-line settings for selected patients with advanced NSCLC, with encouraging outcomes seen in patients with stage III NSCLC. For locally advanced/metastatic NSCLC, immunotherapy, either alone, chemotherapy, and targeted therapies for epidermal growth factor receptor (EGFR), ALK, and ROS1 have remained the standard of care for frontline treatment. Molecular profiling and immunotherapy have been incorporated into advanced disease settings, expanding treatment options, improving survival outcomes, and setting the stage for more personalized care.

Due to the unique properties of cancer immunotherapy and the rapid advancements in this field, clinical guidance for the use of these medications is essential, including appropriate patient selection, treatment sequencing, monitoring of response, management of adverse events, and biomarker testing [48], photothermal therapy [49] and nanotechnology-mediated drugs [50] are emerging strategies for the treatment of lung cancer. Additionally, the availability of new therapy is associated with an increased survival rate among NSCLC patients [51]. SCLC [52] is a highly aggressive cancer characterized by early metastasis, high growth rate, rapid drug resistance, and poor survival rates. Compared with NSCLC, more research is needed for SCLC [53].

## Artificial intelligence

### History of AI

**Fig. 1:**
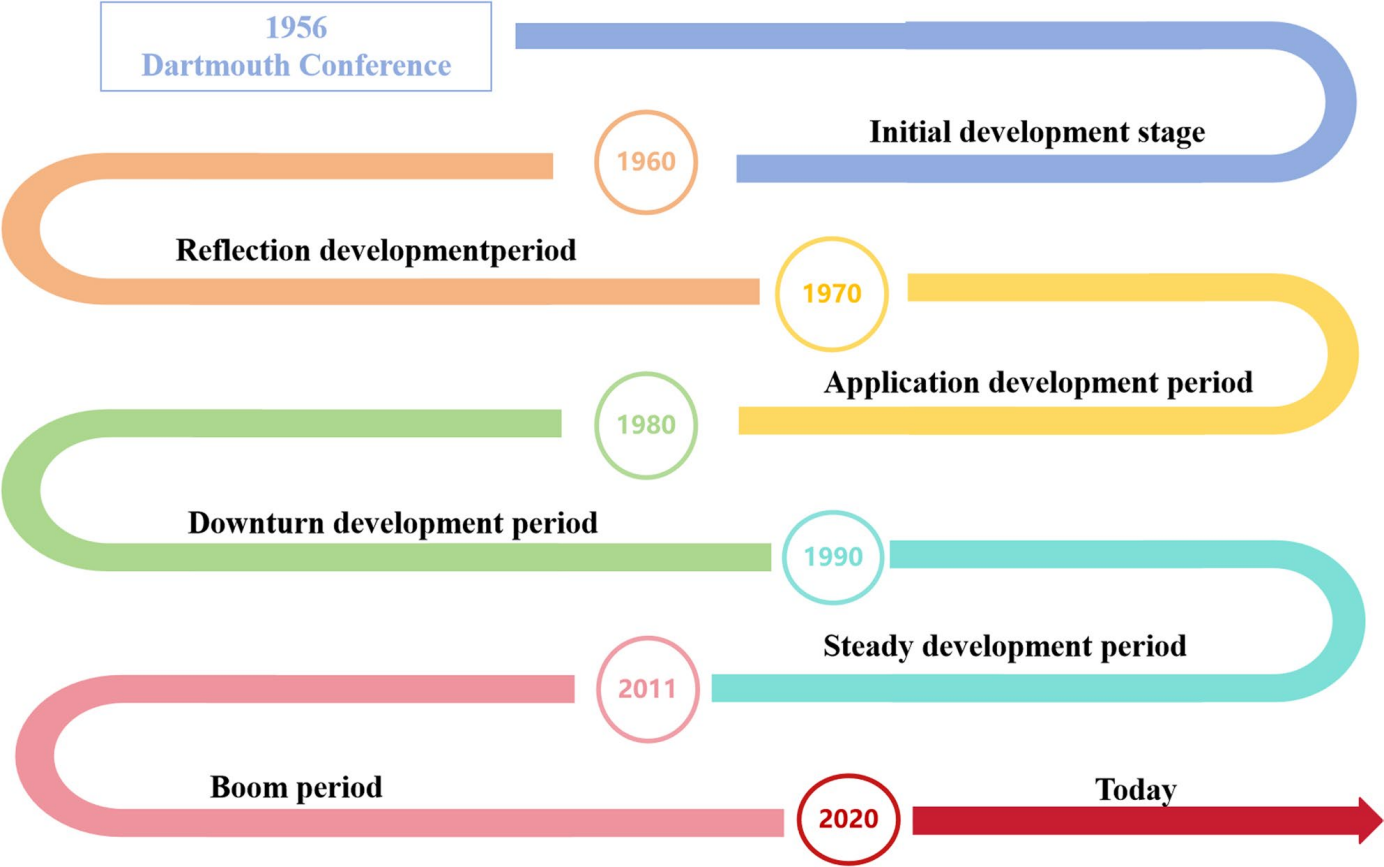
The history of AI development. The history of AI development is long and winding; however, it has achieved significant accomplishments and continues to play an increasingly important role in human life and existence

In the summer of 1956, scientists such as McCarthy and Minsky came together at Dartmouth College in the USA for a conference to discuss “how to simulate human intelligence using machines”. During this meeting, the concept of “Artificial Intelligence” was introduced, marking the birth of the field of AI. During the initial phases of AI development, people’s expectations were overly optimistic, establishing unattainable objectives. Owing to technological limitations and difficulties in practical applications, these goals were not achieved, resulting in a downturn in the progression of AI. The expert systems that emerged in the 1970s simulated the knowledge and experience of human experts to solve problems in specific domains, marking a significant breakthrough in AI as it transitioned from theoretical research to practical application and achieved success in fields such as medicine, chemistry, and geology, propelling AI into a new peak of application development. But, as the scale of AI applications continues to expand, development is gradually slowing. Due to the subsequent development of network technologies, particularly internet technologies, innovative research in AI has prompted the steady development of AI technologies. Subsequently, the rapid advancement of deep learning technology has accelerated innovative research in AI, propelling these technologies further toward practical applications. To date, generative AI and multimodal integration have become the current focal points (Fig. 1).

### AI technology of learning methods

**Fig. 2:**
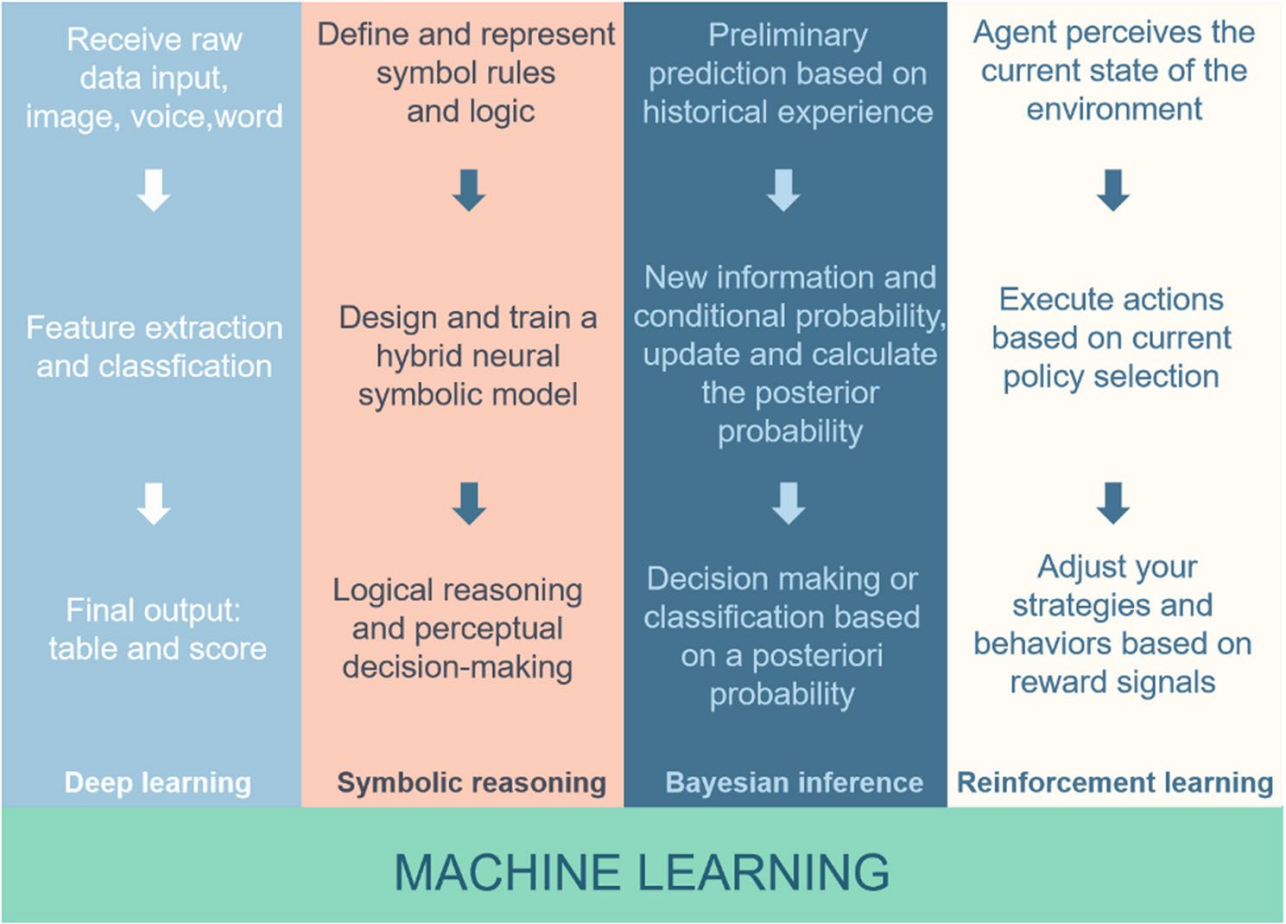
DL, symbolic reasoning, Bayesian inference, and RL are the four core technical directions in the field of AI. They belong to different methodological systems (connectionism, symbolism, probabilityism, and behaviorism). However, they have obvious complementarity in solving complex AI tasks, and the trend of integration is becoming increasingly prominent

AI is a multidisciplinary field of computer science that develops systems capable of simulating human cognitive functions such as learning, reasoning, and decision-making. AI improves its performance through four major learning paradigms: deep learning (DL), Bayesian inference, symbolic reasoning, and reinforcement learning (RL) [54] (Fig. 2).

#### Deep learning (DL)

Deep learning approaches are a subset of machine learning techniques that can recognize intricate patterns within extensive datasets. Machine learning tasks are generally divided into two primary types: supervised learning and unsupervised learning. In the context of supervised learning, the objective is to predict the label (classification) or the response (regression) of each data point using a given set of provided, labeled training examples. In unsupervised learning, such as clustering and principal component analysis, the goal is to learn the patterns inherent within the data itself [55]. DL has led to tremendous progress in many fields. These methods include tumor pathology [56], automated image analysis [57], diagnosis [58], and coronary angiography [59]. Litjens et al. [60] assessed the role of deep learning in histopathological examinations and verified its exceptional performance in identifying prostate cancer and detecting breast cancer metastasis. Ertosun and Rubin [61] suggested an automated method for grading gliomas using deep learning. Additionally, in thyroid cancer [62], they have investigated the potential of a deep learning-based system for the automated prediction of microsatellite instability directly from color whole slide images stained with hematoxylin and eosin (H&E)-stained whole-slide images (WSIs) [63].

#### Symbolic reasoning methods

Symbolic reasoning is the process of abstracting real-world problems into symbols and rules and solving problems through the manipulation of these symbols, similar to playing a ‘symbol game’ with logical rules. As long as the rules are correct, the results of the reasoning can accurately reflect the real situations. This method allows complex problems to be standardized and formalized, making it easier for people to quickly understand and resolve issues. Zhongyi et al. [64] proposed a symbolic logical model of thought that utilizes metainterpretive learning to induce causal effects between spinal diseases and to uncover valuable pathogenic factors useful for diagnosing spinal diseases based on pathogenesis.

#### Bayesian inference

Bayesian inference provides a statistical approach to updating the probability of a hypothesis’s truth, taking into account prior beliefs and the latest evidence. The evidence for the hypothesis is marginalized by the probability of observing the evidence without any assumption [65, 66]. Everett et al. [67] applied Bayesian strategies to a population inference case and an individual patient’s parameters. A basic application in the case of a patient-specific Bayesian inference was demonstrated via a case study, and then, tumor heterogeneity was inferred and quantified.

#### Reinforcement learning

Reinforcement learning, initially a field in computer science, focuses on learning through trial and error to gain rewards or evade penalties [68]. Typically, reinforcement learning algorithms are restricted to discovering a single solution for a particular task, even though various solutions may be available [69, 70]. Lingrui et al. [71, 72] confirms the benefit of reinforcement learning from AI feedback for impression summarization, paving the way for the application of AI feedback in clinical CT report summarization. This work will serve as the basis for the future development of multimodal large language models in the field of radiology. Additionally, reinforcement learning has been explored for the hemodynamic optimization of septic patients in the intensive care unit [73]. Xuan et al. [74] presented MedRIA, a medical reinforcement learning inquiry assistant, has been developed to aid the medical research process by skillfully managing inquiries, assessing symptoms, recommending examinations, and other functions.

### Application of AI in medicine

In recent years, AI has been employed in numerous medical fields, such as diagnostic applications in radiology [75], anesthesiology [76], and pathology [77] to therapeutic applications. The applications of AI encompass the detection and classification of cancer, the analysis of the molecular characteristics of tumors and their microenvironment, the discovery and repurposing of drugs, as well as the prediction of therapeutic outcomes for patients and personalized treatments.

Watson for Genomics (WG) is a cognitive computing technology that helps physicians analyze and manage patients’ genomic maps by acquiring a large volume of data and comparing the relationships between genes and genomes to develop personalized treatment plans [78]. AI models that predict patient responses to other cancer treatments from omics or imaging data have been widely reported. They presented an AI system that is capable of surpassing human experts in breast cancer prediction [79]. Jean-Emmanuel et al. [80] believing that the generalization of electronic health records (EHRs) provides a unique opportunity to create adequate phenotypes, combining EHR and genomics through machine learning could generate high-quality evidence for precision medicine.

## Application to lung cancer

**Fig. 3:**
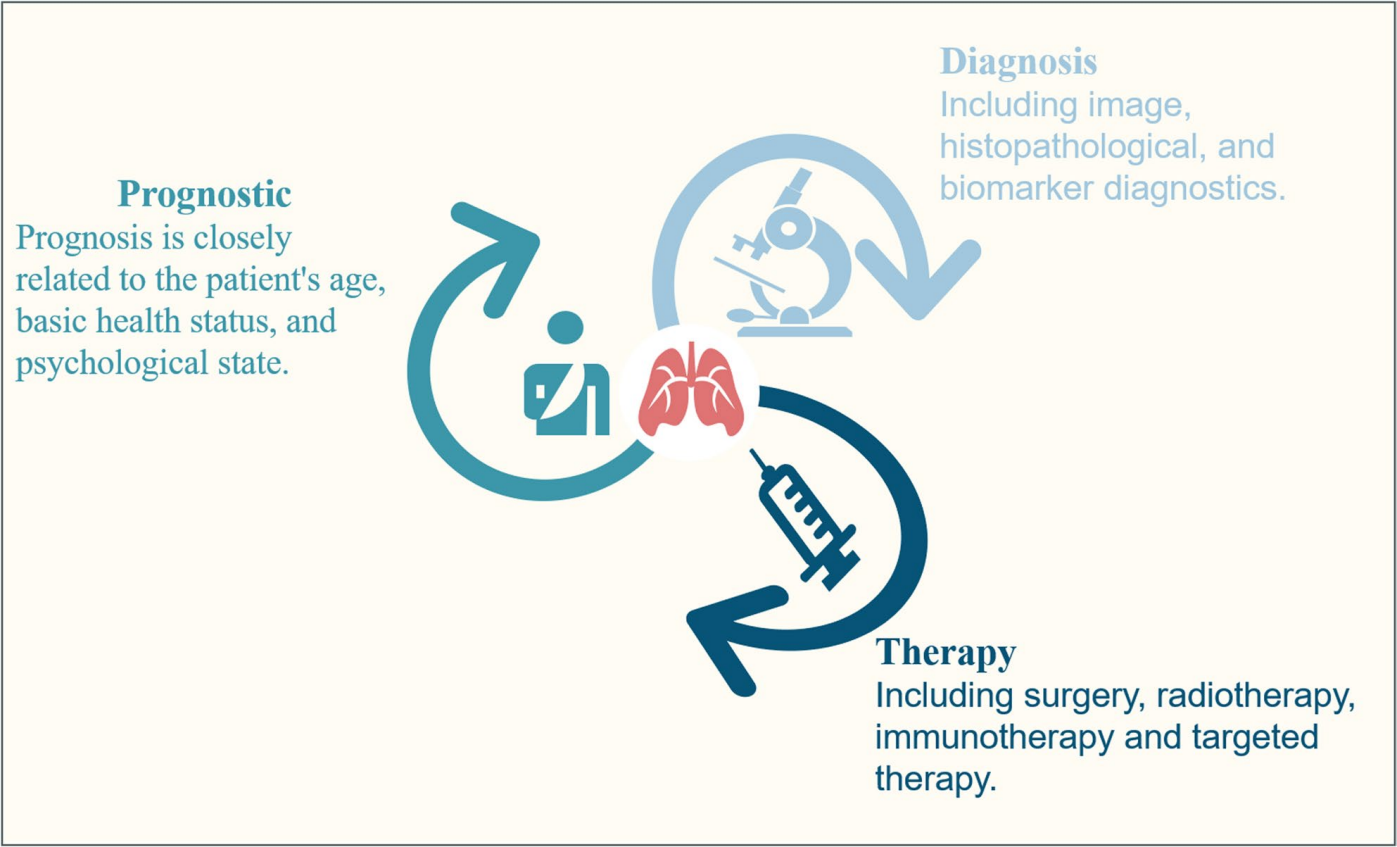
Lung cancer, the cancer with the highest mortality rate, is an aspect that cannot be overlooked in the diagnosis, treatment, and prognosis of patients in the fight against lung cancer

The increasing importance of AI in various domains has sparked widespread adoption in cancer research [81]. The precise identification of individuals at high risk of lung cancer is essential for optimizing lung cancer screening. Researchers have committed to comparing the effectiveness of traditional regression models with that of AI-based models in predicting future lung cancer risks. Compared to traditional regression models, AI-based models, particularly those utilizing imaging data, demonstrate promising approaches to improving the prediction of lung cancer risk [82]. AI has played an important role in tumors, driving breakthroughs in the challenges of cancer treatment. Additionally, it plays an essential role in the prevention, diagnosis, treatment, and prognosis of lung cancer (Fig. 3).

### Diagnosis

AI algorithms are capable of analyzing intricate data, distinguishing lung cancer patients from healthy people, and predicting the likelihood of cancer spreading [83]. Wang et al. [84] developed a prospective risk prediction model that can identify high-risk patients in the general population who may be newly diagnosed with lung cancer in the coming year. They extracted individual patient EHRs for model construction and validation to predict the risk of incident lung cancer. A machine-learning method called ‘lung cancer likelihood in plasma’ (Lung-CLiP) was developed and prospectively validated by Jacob et al. [85], they demonstrated the ability to robustly distinguish early-stage lung cancer patients from risk-matched controls. Taken together, their findings suggest that a lung cancer screening model with AI is capable of identifying factors that are useful for predicting lung cancer via the use of clinical information available 1 year before the clinical diagnosis is made and the effective use of EMRs to identify high-risk individuals for developing lung cancer [86].

Besides pinpointing high-risk groups for lung cancer, it is essential to accurately classify cancer types such as small cell carcinoma, adenocarcinoma, and squamous cell carcinoma. In their study, Mingsi et al. [87] used a meta-analysis and a substantial amount of recent data to determine AI’s diagnostic value for lung cancer, revealing significant accuracy. Lay Teng’s team also performed this work [88]. According to the authors, AI is highly valuable for diagnosing lung cancer and is more feasible for broader application in clinical diagnosis. Currently, there are three common typical diagnostic methods include image, histopathological, and biomarker diagnostics.

#### Imaging diagnostic

There are quick advancements in the growing area of biomedical informatics and information technology [89]. One cannot overlook the advancements in medicine amidst technological progress, including how AI enhances diagnostic efficiency through medical imaging recognition, such as lung CT nodule detection. A large amount of data will be generated during the diagnosis [90], such as CT [91], MRI [92], and PET [93]. In formulating plans, doctors typically subjectively assess data based on their experience. However, the visual features of image data observed by doctors with the naked eye are often incomplete, leading to many critical aspects of image data being overlooked. In recent years, many researchers have attempted to utilize mathematics and algorithms to extract quantitative information to support the diagnosis of cancer progression [94].

AI’s role can evaluate screening eligibility, cut down radiation exposure, improve image clarity for the detection and classification of lung nodules, and decide on the most suitable screening intervals with LDCT [95]. Bihong T et al. [96] introduced a CT radiomic model featuring a neural network classifier that effectively differentiates SCLC from NSCLC adenocarcinoma, with an area under the curve (AUC) of 0.93, indicating satisfactory performance. Wang et al. [97] present an innovative AI-driven approach that enhances nodule risk stratification in the Chinese population and, when combined with mobile CT units, has the potential to expand LC screening coverage. A Lung-RADS system was developed in the US to stratify lung nodules into categories of increasing risk of LC which have the potential to reduce unnecessary biopsies and improve time to diagnosis. M S Kavitha et al. [98] proposed a highly efficient classification model for cancer staging diagnosis, which processes CT lung images and demonstrates high imaging sensitivity and resolution in distinguishing lung nodules. Atsushi et al. [99] proposed an automatic classification system for lung cancer in microscopic images has been developed using deep convolutional neural networks. According to the evaluation results, the accuracy of correctly classifying images is approximately 70%. They demonstrate that the use of the AdaBoost algorithm for image classification and segmentation can significantly aid in the future diagnosis of lung cancer and spinal bones [100]. These results indicate that AI greatly promotes the speed and accuracy of imaging diagnosis for classifying lung cancer.

#### Histopathological diagnosis

The examination of histopathological sections is one of the primary methods employed by pathologists to assess the status of lung tumors [101]. Xiaoxi et al. [102] has established an instrument called ANORAK (pyrAmid pooliNg crOss stReam Attention networK), which is capable of precisely quantifying the growth patterns of lung adenocarcinomas by illustrating the morphological changes within the tumor through the representation of cross-sectional patterns stained with hematoxylin and eosin. Javier et al. [103] has designed a classifier for a diagnostic support system for the detection of non-small cell lung cancer. This classifier utilizes AI technologies to automatically classify pathological images of lung tissue into adenocarcinoma, squamous cell carcinoma, and healthy tissue, achieving an accuracy rate of 97.11% to 99.69%. Another team trained a deep convolutional neural network (inception v3) on whole-slide images obtained from The Cancer Genome Atlas to classify them accurately and automatically into LUAD, LUSC, or normal lung tissue. The performance of the method is comparable to that of pathologists, with an AUC of 0.97 [104]. Zhao et al. [105] proposed a deep learning solution that classifies models of histopathological subtypes of nonmucinous lung adenocarcinoma, which can be automatically classified. The application of AI in histopathological diagnosis can increase the speed and accuracy of diagnoses made by pathologists.

Recently, the development of robotic platforms and devices for bronchoscopy has seen significant progress, with systems such as the Monarch Platform15 and the Ion Endoluminal System16 leading the way. They combines slimmer robotic bronchoscopes with AI-driven planning systems for nodule segmentation and 3D airway mapping, have been introduced to enhance manoeuvrability and stability during lesion sam-pling. Zhang et al. [106] presented a low-cost comprehensive AI co-pilot bronchoscope robot to improve the safety, accuracy, and efficiency of broncho-scopic procedures. Notably, the AI–human shared control algorithm maintains better bronchus centring and exhibits lower operation errors than an expert operator. It is conducive to the pathological diagnosis.

#### Biomarker diagnosis

Biomarkers are often used for selecting cancer treatments and prognosis, and they can also assist in preliminary diagnosis and staging prediction through AI. AI has emerged as a promising tool for identifying the EGFR mutation status from digital pathology images. The meta-analysis by the team assessed the diagnostic precision of AI models in forecasting EGFR mutations using WSIs in individuals with lung cancer [107]. Nan et al. [108] proposed solid connection between the TIIC signature score and tumor immunity as well as metabolism has been revealed. Additionally, the TIIC signature score, a newly demonstrated biomarker, also showed a higher prognostic value than age, gender, and TNM staging system. Mehran et al. [109] identified 255,393 NSCLC-specific oncRNAs from The Cancer Genome Atlas (TCGA) smRNA-seq database and proposed a generative AI model, which enhances the performance of for early cancer detection and tumor subtyping. Dong et al. [110] proposed a multichannel and multitask deep learning model based on CT images, which can simultaneously predict the mutation status of EGFR and KRAS. AI models are now essential in lung cancer screening, providing advantages such as reducing radiation exposure, precisely identifying and classifying lung nodules, and tailoring screening schedules.

### Therapy

In recent years, oncology has witnessed significant advancements, including the emergence of molecular targeted therapies, cancer stem cell molecules, and personalized immunotherapy, as well as genomic analyses for stratified diagnosis and treatment. The progress of these therapeutic methods has enhanced the arsenal of tools available to combat cancer. The application of AI will facilitate the collection and compilation of information, assisting physicians in making the most informed treatment decisions for patients.

#### Surgery

For early-stage lung cancer, the best treatment option is surgical resection [111]. The application of AI and machine learning (ML) supports surgical decisions by better recognizing detailed and complex anatomical features [112]. All these advancements have led to faster recovery and fewer complications in patients. With future advancements such as AI-driven automation, nanorobots, microscopic incision surgeries, semiautomated telerobotic systems, and the impact of 5G connectivity on remote surgery [113–115], the growth curve of robotic surgery signifies innovation [116].

Virtual biopsy via AI may help select the best surgical time for subsolid nodules [117]. Yujin et al. [118] analyzed high-resolution CT images from 472 patients with stage I NSCLC to evaluate the efficiency of AI in detecting intrathoracic pleural infiltrations in lung cancer patients, hoping to improve the preoperative diagnostic outcomes for NSCLC patients. Siavash et al. [119] developed two machine learning prediction models to assess and identify high-risk patients with thoracic vertebral complications who are not suitable for lung segment resection. Currently, surgery remains the most commonly used method for curing early-stage lung cancer. AI can support surgical treatment by assisting surgeons in making timely diagnoses to mitigate the negative impact of postoperative complications on patients. However, AI technology still faces huge problems in the development of surgical treatment application, including ethics and accuracy rate.

#### Radiotherapy

Radiotherapy is also one of the primary modalities for tumor treatment, commonly used in conjunction with other methods. The diagnosis before radiotherapy and the mid-treatment assessments rely on a substantial amount of clinical and imaging data. Most of this data is stored in electronic tumor information systems, which can be integrated with AI to assist clinicians and patients in making treatment and diagnostic decisions, including predicting treatment outcomes and categorizing risk groups [120]. Peimeng et al. [121] developed an LCDigital RT prediction tool that utilizes heat maps to visualize the primary tumor area, assisting physicians in formulating adjuvant radiotherapy plans, thereby reducing the occurrence of radiotoxicity events during treatment and enhancing the therapeutic efficacy of radiotherapy. Wenmin et al. [122] employed the developed AI model based on the PRISMA guidelines to predict the response of lung cancer patients following radiotherapy. AI models can predict prognosis outcomes of lung cancer patients after radiotherapy. Nika et al. [123] calculated 218 dose fractions for 10 patients to assess the inter-fraction stability of dose distribution delivered by two different radiotherapy methods. Radiation pneumonitis (RP) is a common complication following radiotherapy in lung cancer patients. Fengsong et al. [124] analyzed 77 cases of locally advanced squamous cell lung cancer (LASCLC) patients undergoing volumetric modulated arc therapy (VMAT) synchronous radio-chemotherapy, proposing a machine learning model that can predict the occurrence of RP in lung cancer patients after receiving VMAT. Thus, AI can assist physicians in evaluating treatment plans before radiotherapy, further reducing the likelihood of adverse reactions post-treatment [115].

#### Immunotherapy

Immunotherapy is also one of the commonly used treatment modalities for lung cancer, providing an innovative and revolutionary approach in the fight against cancer. Various immunotherapeutic methods, such as immune checkpoint inhibitors (ICIs), cancer vaccines, and cell-mediated immunotherapy, are capable of treatment for lung cancer [125]. But in most cases, the immune response to tumors in cancer patients is gradually diminished, allowing cancer cells to bypass immune system checks [126].

Sun et al. [127] developed a radiomic feature of CD8 T cells that can predict the efficacy of anti-PD-1 or anti-PD-L1 immunotherapy. Trebeschi et al. [128] also constructed a radiomic feature that can predict the response to immunotherapy in lung cancer patients. In another study, combining deep learning with radiomic features can predict the response of patients with advanced NSCLC to immunotherapy, achieving an AUC of 0.960 [129]. Maliazurina et al. [130] also developed a deep learning CT feature that successfully predicts the survival of NSCLC patients undergoing immune checkpoint inhibitor treatment, thereby assisting physicians and patients in selecting appropriate immune checkpoint inhibitors to enhance treatment efficacy. Immunotherapy represents a hopeful new method for treating NSCLC. Furthermore, combining AI with multiomics data can facilitate the integration of diverse data types and enable automated predictions, ultimately offering personalized cancer treatment for patients [131].

#### Target therapy

Over the past decade, the treatment paradigm for NSCLC has undergone significant changes, particularly with the emergence of molecular targeted therapy as an important treatment approach for lung cancer. A variety of molecular targeted drugs for lung cancer treatment have been developed [132]. With lung cancer treatment increasingly guided by biomarkers and the swift development of effective targeted therapies, organizations have tried to establish best practices [133]. In lung cancer, it is common for major cell signaling and regulatory pathways to be altered due to either overexpression or changes in gene sequences [8, 51].

Currently, the EGFR pathway is a key target in NSCLC. Lu et al. [134] presented LUCID, a multimodal AI framework that integrates multimodal data to non-invasively predict EGFR mutations and survival outcomes in lung cancer patients. Wang et al. [135] proposed a fully automated artificial intelligence system (FAIS) that can predict EGFR genotype and the prognosis of EGFR-TKI treatment through CT imaging and EGFR gene sequencing. Zhou et al. [136] proposed a machine learning-aided drug screening for selective inhibitors. Seo et al. [137] discussed key therapeutic drugs for EGFR exon 20 insertions, such as amivantamab, mobilocertinib, and sunvozertinib, which show great promise in the treatment of NSCLC. Additionally, PD expression on tumors is a predictive biomarker for sensitivity to immunotherapy [138]. Luo et al. [139] performed extensive experiments and proposed a collaborative filtering technique that is both computationally efficient and cost-effective, which can identify the compounds that are most suitable for patients without genetics. In addition, there are many immunotherapy targets. Through the combination with AI technology, we can select more extensive, rapid, and cost-effective targeted genes with better therapeutic effects.

### Prognostic

The prognosis of NSCLC depends upon the extent of disease (stage) and performance status. An individual tumor is often heterogeneous, and its various features can be visualized noninvasively via medical imaging. Some of these tumor features have prognostic value across cancer types.

Kenneth et al. [140] analyze radiology report text to identify clinical turning points and could reliably forecast prognosis and alterations in systemic therapy for cancer patients. Aerts et al. [141] extracted radiomic features from computed tomography images from a large dataset of 1,019 lung and head and neck cancer patients and reported that many radiomic features have prognostic power. Hind M et al. [142] emphasizing the significant potential of AI models in facilitating the non-invasive assessment of lung cancer prognosis and predictive biomarkers, which can enhance diagnostic accuracy and guide the selection of treatment methods. Daniel et al. [143] conducted a comprehensive and high-throughput computational analysis to identify prognostic histological characteristics in lung squamous cell carcinomas. Based on the screening results, they created and validated an H&E biomarker for tissue microarrays that doesn’t need immunohistochemistry or special software, and also developed an H&E stromal inflammation (SI) score. Luo et al. [134] proposed that LUCID’s risk predictions also could assist clinicians in treatment planning by providing objective prognostic information. As stated above, enhancing the prognosis detection and accuracy analysis of lung cancer through AI technology can provide strong support for future research or treatment of lung cancer. The use of AI in personalized oncology has the potential to significantly improve patient outcomes in lung cancer care.

## Conclusion and future

The rapid development of technology has led to significant progress in lung cancer research, bringing us closer to the ‘truth’ of the occurrence and development of cancer. AI plays a prominent role in this field. However, most current achievements in lung cancer research have not yet been applied clinically and have failed to alleviate the suffering of cancer patients.

The translation of lung cancer research findings into practical applications relies heavily on the advancement of AI technologies. The primary reason is that AI requires massive amounts of data, and collecting more data remains a major challenge for AI development. We need to explore ways to reduce costs more effectively, comply with regulations, and utilize simpler technologies to collect large datasets for model training, testing, and validation. Second, the accuracy of AI across different devices and populations remains unsatisfactory. Improving accuracy and stability through technological upgrades is another critical issue that AI must address in the medical field. Currently, external validation and clinical implementation of AI in lung cancer research are limited [144]. Additionally, most AI models operate as “black boxes,” making it difficult to clearly explain diagnostic reasoning to doctors or patients, which affects clinical trust. To address these issues, the most crucial first step is to popularize early screening and accurately incorporate large amounts of patient information into diagnosis, treatment, and prognosis.

Precision medicine has propelled the diagnosis and treatment of lung cancer. Nevertheless, the survival time of patients remains to be extended, and the quality of life of advanced-stage patients requires improvement. There are several underlying reasons. Firstly, the mechanistic research of lung cancer remains inconclusive. Although numerous researchers have investigated the mechanisms of lung cancer development and progression, further in-depth exploration is still necessary. Secondly, challenges exist in the translation from research findings to clinical applications. At present, a substantial amount of scientific research achievements cannot be effectively utilized in the clinical diagnosis and treatment of lung cancer. Many anti-lung cancer drugs often fail in the third phase of clinical trials. Finally, the side effects of drugs have a detrimental impact on patients’ survival time. During the late-stage treatment of lung cancer, patients frequently succumb to drug side effects, such as neurasthenia, meningitis, and impairment of liver and kidney functions. These side effects not only significantly shorten patients’ survival time but also reduce their quality of life. The appropriate application of AI technology can help identify at an early stage drugs that are unlikely to be successful in clinical practice. Moreover, AI has the potential to help doctors make more informed decisions regarding drug use during diagnosis, thereby alleviating patients’ suffering and enhancing their quality of life. Currently, there is a pressing need to further enhance the application of AI in the diagnosis, treatment, and prognosis of lung cancer. In the future development of diagnostic, therapeutic, and prognostic processes, AI should actively integrate radiogenomics and clinical data to dynamically optimize targeted therapy or immunotherapy regimens, ultimately forming a closed-loop intelligent system encompassing “screening-diagnosis-treatment-rehabilitation”.

By applying AI technology to areas where it is most needed in human healthcare, we can make significant progress in addressing human diseases and contribute to a more prosperous and fulfilling life for humanity.

## Data Availability

No datasets were generated or analysed during the current study.

## Abbreviations

AI: Artificial Intelligence
SCLC: Small cell lung cancer
NSCLC: Non-Small Cell Lung Cancer
LUAD: Lung adenocarcinoma
LUSC: Lung squamous cell carcinoma
ES-NSCLC: Early-stage non-small cell lung cancer
LDCT: Low-dose CT
MRI: Magnetic Resonance Imaging
CT: Computed tomography
PET: Positron emission tomography
RT: Radiation Therapy
SABR: Stereotactic ablative radiotherapy
DL: Deep Learning
RL: reinforcement learning
WG: Watson for Genomics
HER: Electronic health records
WSIs: whole-slide images
IT: Information Technology
AUC: Area under the curve
ANORAK: pyrAmid pooliNg crOss stReam Attention networK
FCM: Fuzzy C-Means Clustering
SVM: Support Vector Machine
SI: Stromal Inflammation
ML: Machine Learning
RP: Radiation pneumonitis
VMAT: Volumetric modulated arc therapy
LASCLC: Locally advanced squamous cell lung cancer
ICIs: Immune Checkpoint Inhibitors
TKs: Tyrosine Kinases
TKIs: Tyrosine kinase inhibitors
EGFR: Epidermal growth factor receptor
PD-L: Programmed death-ligand 1
